# Overexpressed Proteins in HCC Cell-Derived Exosomes, CCT8, and Cofilin-1 Are Potential Biomarkers for Patients with HCC

**DOI:** 10.3390/diagnostics11071221

**Published:** 2021-07-06

**Authors:** Hyo Jung Cho, Geum Ok Baek, Moon Gyeong Yoon, Hye Ri Ahn, Ju A Son, Soon Sun Kim, Jae Youn Cheong, Jung Woo Eun

**Affiliations:** 1 Department of Gastroenterology, Ajou University School of Medicine, Suwon 16499, Korea; pilgrim@ajou.ac.kr (H.J.C.); ptok99@hanmail.net (G.O.B.); ymk8028@hanmail.net (M.G.Y.); rhkwp37@naver.com (H.R.A.); gracia33781@gmail.com (J.A.S.); 105547@aumc.ac.kr (S.S.K.); 2 Department of Biomedical Sciences, Ajou University Graduate School of Medicine, Suwon 16499, Korea

**Keywords:** hepatocellular carcinoma, exosome, proteomics, cofilin-1, CCT8

## Abstract

Protein markers of hepatocellular carcinoma (HCC)-derived exosomes (HEX) have not yet been fully evaluated. Here, we identified novel protein contents of HEX and their clinical significance as biomarkers. Exosomes were isolated from human HCC cell lines and an immortalized normal hepatocyte cell line. Proteomic analyses revealed 15 markedly overexpressed proteins in HEX. The clinical relevance of the 15 proteins was analyzed in public RNA-sequencing datasets, and 6 proteins were selected as candidate of potential biomarkers. Serum CCT8 and CFL1 were markedly overexpressed in test cohort (*n* = 8). In the validation cohort (*n* = 224), the area under the curve (AUC) of serum CCT8 and CFL1 for HCC diagnosis was calculated as 0.698 and 0.677, respectively, whereas that of serum alpha-fetoprotein (AFP) was 0.628. The combination of three serum markers (CCT8, CFL1, and AFP) demonstrated the highest AUC for HCC diagnosis. (AUC = 0.838, 95% confidence interval = 0.773–0.876) Furthermore, higher serum CCT8 and CFL1 concentrations were significantly associated with the presence of vascular invasion, advanced tumor stage, poor disease-free survival, and poor overall survival. Cofilin-1 and CCT8, enriched proteins in HEX, were identified as potential diagnostic and prognostic serum biomarkers for HCC patients.

## 1. Introduction

Hepatocellular carcinoma (HCC) accounts for 80%–90% of primary liver malignancies and is the sixth most common malignancy worldwide [1,2]. Although therapeutic strategies for HCC have continually advanced over the past several decades, the overall prognosis of HCC still remains very poor with an estimated 5-year survival rate of 32.8% [3]. The development of precision medicine based on reliable biomarkers is anticipated to improve the prognosis of HCC.

Cancer tissue acquisition is a key process for obtaining genomic or proteomic information about the cancer. Recently, liquid biopsy has emerged as a promising technology in cancer science for detecting tumor-derived molecules by minimally invasive methods [4]. Considering the cost and complication risks for tissue biopsies, liquid biopsy has significant advantages over solid tissue biopsy. Exosomes are one of the most important parts of liquid biopsy [5,6]. Exosomes are small extracellular vesicles of endocytic origin about 30–100 nm in size. They enclose genetic materials of the parent cells [7]. Exosomes deliver various classes of molecules, including nucleic acids, metabolites, and proteins from the parent cell to a recipient cell. Thus, exosomes are considered as a key player in cell-to-cell communication [8]. Many studies have been performed to identify the molecular contents of exosomes for finding biomarkers and therapeutic targets. Proteins commonly found in exosomes have been designated as “exosome-specific” markers, including CD9, CD63, and CD81 [7]. Increased CD63-positive exosomes in patients with cancer are reported; therefore, CD63 is suggested as a cancer biomarker for diagnosis and prognosis [9]. In the field of HCC, specific proteins of HCC cell-derived exosomes (HEX) and their clinical role have not been evaluated yet.

In this study, we identified specific protein contents of HEX by performing proteomic analyses. Furthermore, the clinical significance of the identified specific proteins in HEX was evaluated in an independent liver disease cohort.

## 2. Materials and Methods

### 2.1. HCC Cell Lines and Culture

HCC cell lines, including Hep3B and Huh-7 (Korean Cell Line Bank, Seoul, Korea), were cultured with Dulbecco’s Modified Eagle Medium (DMEM, Gendepot, Katy, TX, USA) supplemented with 10% fetal bovine serum (FBS, Gendepot) in a humidified atmosphere with 5% CO_2_. Immortalized normal hepatocytes, THLE-2 (ATC, Manassas, VA, USA), were cultured in a LHC8 medium (Gibco, Rockville, MD, USA) containing 70 ng/mL phosphoethanolamine (Sigma, St. Louis, MO, USA), 5 ng/mL epidermal growth factor (EGF, Sigma), 10% FBS, and antibiotics at 37 °C in a humidified atmosphere with 5% CO_2_. 

### 2.2. Exosome Purification from Cell Culture Media and Patients’ Serum

On achieving 90% confluency, cells were washed three times with phosphate-buffered saline (PBS) and supplemented with DMEM containing 10% exosome-depleted FBS (System Biosciences, Palo Alto, CA, USA) during 72 h. Thereafter, cultured medium was collected and centrifuged consecutively at 300× *g* for 10 min at 4 °C, at 2000× *g* for 10 min at 4 °C, and then at 7500 rpm for 20 min at 4 °C for removing cells, dead cells, and cell debris. Further, the supernatants were harvested and ultra-centrifuged at 30,000 rpm for 70 min at 4 °C to harvest pellets of crude exosomes. Pellets were washed twice with PBS, resuspended in 100 µL PBS, and stored at −80 °C. For detection of serum exosomal RNA expression, serum exosomal RNA was isolated from 300 µL serum using ExoQuick (System Biosciences).

### 2.3. Transmission Electron Microscopy

For imaging analyses, 10 nm gold-conjugated anti-CD63 antibody staining to exosomes was performed. Sample fixation was performed with 2% glutaraldehyde and 4% paraformaldehyde for two hours at room temperature. Thereafter, visualization of exosomes was performed using a transmission electron microscope (TEM, Sigma 500, Carl Zeiss, Jena, Germany).

### 2.4. Western Blot Analysis

Exosomes or cell lysates were denatured in 4X Laemmli sample buffer (Gendepot) and subjected to SDS/PAGE, and proteins were transferred to a PVDF membrane (Merck Millipore, Darmstadt, Germany). Subsequent to immunoblotting with antibodies, including mouse anti-CD63 (ab59479, Abcam, Cambridge, UK) and rabbit anti-LAMP-1 (ab24170, Abcam), proteins were visualized on a Bio-Rad ChemiDoc MP Imager system (Bio-Rad Laboratories, Berkeley, CA, USA).

### 2.5. Two-dimensional Gel Electrophoresis

Two-dimensional gel electrophoresis (2-DE) was carried out as previously described [10]. Aliquots in sample buffer (7 M urea, 2 M thiourea, 4.5% CHAPS, 100 mM dithioerythritol, 40 mM Tris, pH 8.8) were used to immobilize pH 3–10 non-linear gradient strips (Amersham Biosciences, Uppsala, Sweden). Isoelectric focusing was performed at 80,000 Vh. The second dimension was analyzed for 9%–16% linear gradient polyacrylamide gels (18 cm × 20 cm × 1.5 mm) at constant 40 mA per gel over five hours. Protein fixation was done in 40% methanol and 5% phosphoric acid for one hour. Gels were stained with Coomassie brilliant blue G-250 for 12 h. The gels were de-stained with H2O and scanned in a GS710 densitometer (Bio-Rad, Richmond, CA, USA), and the acquired data were converted into electronic files and analyzed with the Image Master Platinum 5.0 image analysis program (Amersham Biosciences, Little Charfent, UK). 

### 2.6. Liquid Chromatography (LC)–Mass Spectrometry (MS)/MS For Peptides Analysis

Nano LC-MS/MS analysis was performed with an Easy n-LC (Thermo Fisher, San Jose, CA, USA) and an LTQ Orbitrap XL mass spectrometer (Thermo Fisher) equipped with a nano-electrospray source. Samples were separated on a C18 nanopore column (150 mm × 0.1 mm, 3 μm pore size; Agilent Technologies, Santa Clara, CA USA). The mobile phase A for LC separation was 0.1% formic acid and 3% acetonitrile in deionized water, and the mobile phase B was 0.1% formic acid in acetonitrile. The chromatography gradient was designed for a linear increase from 0% B to 60% B in 9 min, 60% B to 90% B in 1 min, and 3% B in 5 min. The flow rate was maintained at 1.8 uL-/min. Mass spectra were acquired using data-dependent acquisition with a full mass scan (380–1700 m/z) followed by 10 MS/MS scans. For MS1 full scans, the orbitrap resolution was 15,000 and the automated gain control (AGC) was 2 × 10^5^. For MS/MS in the LTQ, the AGC was 1 × 10^4^. 

### 2.7. ID Mapping and Gene Ontology (GO) Enrichment Analysis

To gain more insight into molecular mechanisms, the protein GI accession numbers from the protein sequence database were uploaded to the Database for Annotation, Visualization, and Integrated Discovery (DAVID) 6.7 Bioinformatics Resources (https://david.ncifcrf.gov/conversion.jsp, accessed on 01 April 2020). To investigate gene signatures that were enriched from known molecular databases, we uploaded our Hep3B or Huh-7 signatures to MSigDB (http://software.broadinstitute.org/gsea/msigdb, accessed on 1 April 2020) at the Broad Institute Gene Set Enrichment Analysis (GSEA) (http://www.broadinstitute.org/gsea, accessed on 1 April 2020). GSEA was conducted by computing overlaps with gene ontology (GO) biological process (BP). Genes in gene set (K), genes in overlap (k), k/K ratio, and *p*-value were used to rank the pathways enriched in each phenotype.

### 2.8. Publicly Available Genomic Data Analysis

To evaluate the expression level of candidate genes, RNA-sequencing data were obtained from The Cancer Genome Atlas Liver Hepatocellular Carcinoma (TCGA-LIHC), International Cancer Genomic Consortium (ICGC), and the Gene Expression Omnibus (GEO) database of the National Center for Biotechnology Information (NCBI) (Accession number: GSE77314) project. RNA expression of the three RNA-seq datasets were base 2 logarithm (log2) transformed (log2 (FPKM + 1)).

### 2.9. Quantitative Real-Time PCR (qRT-PCR)

Serum exosomal RNA was reverse transcribed using the miScript II RT kit (Qiagen, Hamburg, Germany). Further, qRT-PCR was performed using amfiSure qGreen Q-PCR Master Mix (Gendepot) and monitored in real time using an ABI 7300 Real-Time PCR System (Applied Biosystems, San Francisco, CA, USA). The 2-ΔΔCT calculation was used to determine target gene expression relative to the internal control gene, HMBS [11]. All measurements were performed in triplicate. Primer sequences used in the study are listed in Table S1. 

### 2.10. Enzyme-Linked Immunosorbent Assay (ELISA)

An ELISA was used to quantify the levels of six candidate biomarkers in serum. The following commercially available ELISA kits were used: enolase 1 (ENO1) (CSB-E17177h; Cusabio, Houston, TX, USA), CCT8 (MBS2516261), CFL1 (MBS2505977), ANXA5 (MBS704883), HSPB1 (MBS727021), and TPM4 (MBS944560; all previously named kits from Mybiosource Inc., San Diego, CA, USA). The serum samples were diluted at 1:8 and the protocol for the ELISA was performed following the manufacturer’s instructions.

### 2.11. Validation Cohort and Clinical Term Definitions

Serum samples and the data used in this study were provided by the Biobank of Ajou University Hospital, a member of the Korea Biobank Network. The design and procedure of the present study was approved by the Institutional Review Board of the Ajou University Hospital, Suwon, South Korea (AJRIB-BMR-KSP-18-397). The informed consent was waived. Serum samples were collected from patients who visited Ajou University Hospital, Suwon, South Korea between January 2014 and December 2018. The study groups were categorized as normal healthy individuals, patients with chronic hepatitis B (CHB), patients with liver cirrhosis (LC), and patients with HCC. Normal control was defined as patients aged from 18–50 years who visited Ajou Health Promotion Center for a regular health check-up without any medical history and completely normal blood results. Patients with CHB were defined with persistent serum HBsAg for more than six months [12]. Patients with LC were diagnosed based on morphological assessment of an imaging study, liver stiffness in elastography, and blood tests measuring platelet count, albumin level, and international normalized ratio [13]. HCC was diagnosed in patients according to American Association for the Study of Liver Diseases guidelines [14,15]. The clinical data provided contained information about age, sex, etiology of liver disease, aspartate aminotransferase level, alanine aminotransferase level, platelet count, serum alpha-fetoprotein (AFP) level, serum albumin level, serum bilirubin level, and international normalized ratio. Additionally, tumor size, tumor number, presence of vascular invasion, and tumor stage according to modified Union for International Cancer Control (mUICC) staging system were investigated in patients with HCC [16]. Patients at high risk of developing HCC were defined as patients with CHB or LC. Disease-free survival (DFS) was defined as the time from curative treatment to cancer recurrence, whereas overall survival (OS) was defined as the time from HCC diagnosis to death by all causes. 

### 2.12. Statistical Analysis

Data are presented as mean ± standard deviation (SD). Statistical significance of the difference between experimental groups was assessed by paired or unpaired Welch’s *t*-test (* *p* < 0.05, ** *p* < 0.01, *** *p* < 0.001). IBM SPSS software version 22.0 (SPSS Inc., Chicago, IL, USA) and GraphPad Prism version 7.01 software (GraphPad Software, San Diego, CA, USA) were used for statistical analysis. Statistical significance was established at *p* < 0.05. Chi-square test (two-sided) was used to assess the association between categorical parameters. Survival curves were plotted using the Kaplan–Meier method and significant difference between the survival curves was determined using the log-rank test. Receiver operating characteristic (ROC) curves were analyzed to evaluate sensitivity, specificity, and respective area under the curve (AUC) values with a 95% confidence interval (CI) for each candidate biomarker. 

## 3. Results

### 3.1. Isolation of HEX

**Figure 1:**
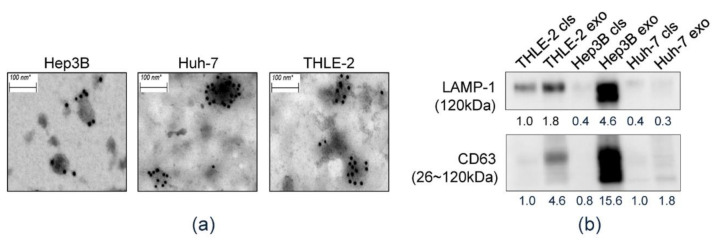
Characteristics of exosomes in liver cell lines. (**a**) Transmission electron microscopic (TEM) images of purified exosomes with 10 nm gold-conjugated anti-CD63 antibody derived from the Hep3B, Huh-7, and THLE-2 cell lines. (**b**) Identifying expression of exosome markers by Western blot analysis.

First, we confirmed the isolated exosomes derived from culture media of each cell line. CD63-immunostained TEM revealed that all isolated samples consisted of CD63-positive spherical vesicles about 30–100 nm in size, which confirmed the efficiency of exosome isolation (Figure 1a). Western blot analysis was performed and CD63 and LAMP-1, which are reported as specific protein markers of exosomes, were detected in exosome preparations (Figure 1b).

### 3.2. Mass Spectrometry Analysis and NGS RNA-seq Data Analysis of HCC-Derived Exosomal Protein Markers

**Figure 2:**
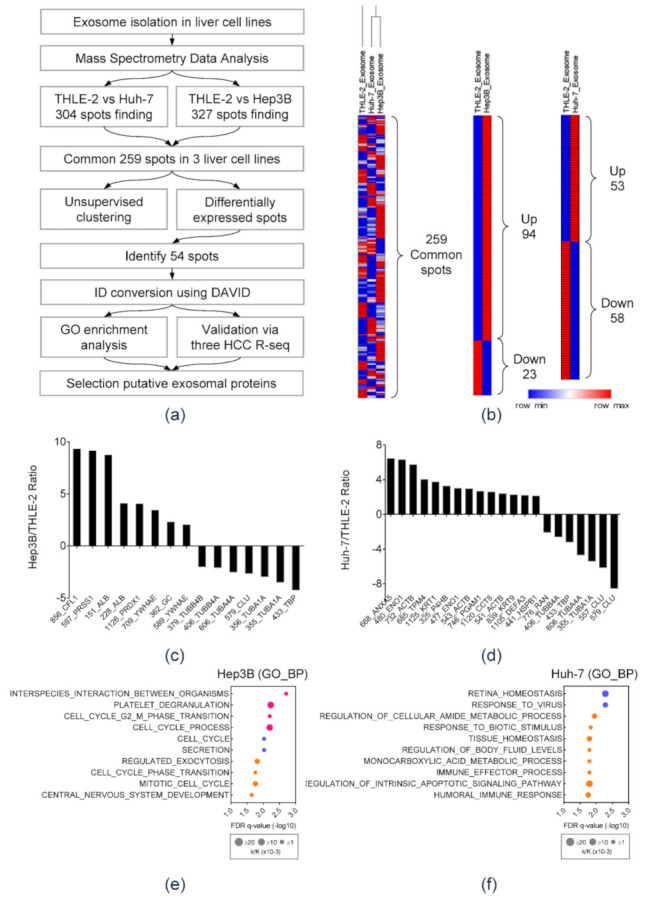
Identification of exosomal protein markers in HCC. (**a**) A schematic view of the procedure to find novel exosomal protein markers in HCC. (**b**) Heat map depicting differentially expressed protein spots in each cell line. (**c**) The expression ratio of differentially identified exosomal proteins in Hep3B as compared to THLE-2. (**d**) The expression ratio of differentially identified exosomal proteins in Huh-7 cell lines as compared to THLE-2. (**e**,**f**) GO enrichment analysis of exosomal proteins specific in (**e**) Hep3B and (**f**) Huh-7 cell lines. The top 10 significantly enriched GO categories were under biological process.

**Figure 3:**
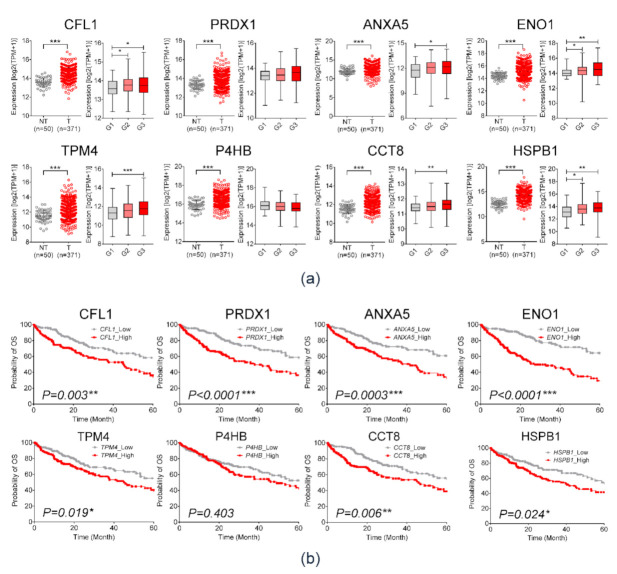
Validation of overexpression of eight exosomal protein markers in hepatocellular carcinoma (HCC) and its clinical relevance through TCGA_LIHC dataset. (**a**) TCGA_LIHC analysis in HCC tissues (*n* = 371) compared with that in non-tumor tissues (*n* = 50) (left), and expression of candidate genes according to tumor histologic grade (right). (Welch’s *t*-test; * *p* < 0.05, ** *p* < 0.01, *** *p* < 0.001) (**b**) Kaplan–Meier plot for overall survival according to mRNA expression of exosomal proteins in TCGA_LIHC. The *p*-value was obtained with the log-rank test.

The flow chart for analysis is illustrated in Figure 2a. Differences between exosomal protein profiles of each cell line were identified via 2-DE assay (Figure S1). Among 259 commonly expressed protein spots, 94 spots were upregulated and 34 spots were downregulated in Hep3B-derived exosomes. In Huh-7-derived exosomes, 53 protein spots were upregulated, whereas 58 spots were downregulated (Figure 2b). With respect to spot intensity ratio, 54 differentially expressed protein spots were selected for proteomic analyses (Supplementary Table S1). Thereafter, protein GI accession numbers were converted using the DAVID program. Figure 2c,d shows the bar chart of up- or downregulated proteins in HEX compared to THLE-2-derived exosomes. Figure 2e,f demonstrates GO enrichment analysis of exosomal proteins specific in Hep3B (e) and Huh-7 (f) cell lines. The top 10 significantly enriched GO categories were under biological process. Fifteen proteins were markedly overexpressed (≥2.5 times) proteins in HEX rather than those of THLE2. (Supplementary Table S2). To select biomarker candidates among the 15 proteins, clinical significance was evaluated using three publicly available RNA-sequencing (R-seq) datasets including TCGA, ICGC, and GSE77314. In each dataset, expression of the 15 candidate genes was compared between HCC and non-HCC tissues (Supplementary Table S2). Eight genes that are commonly overexpressed in HCC tissue in the three R-seq datasets were as follows: cofilin 1 (CFL1), peroxiredoxin 1 (PRDX1), annexin A5 (ANXA5), enolase 1 (ENO1), tropomyosin alpha-4 (TPM4), prolyl 4-hydroxylase (P4HB), chaperonin-containing TCP1 subunit 8 (CCT8), and heat shock protein beta-1 (HSPB1). Expression of the eight candidate genes in the TCGA data is demonstrated in Figure 3a. All eight candidate genes were more significantly overexpressed in HCC tissue than non-tumor tissue (*p* < 0.001), and patients with overexpression of those genes showed significantly poor OS in Kaplan–Meier analysis (Figure 3b). Among them, six candidates, including CFL1, ANXA5, ENO1, TPM4, CCT8, and HSPB1, were positively correlated with histologic grades of HCC (Figure 3a, left panel). Consequently, the following six proteins were selected for further validation studies: CFL1, ANXA5, ENO1, TPM4, CCT8, and HSPB1.

### 3.3. Diagnostic Performance of Serum Exosomal Protein Markers in the Test Set

**Figure 4:**
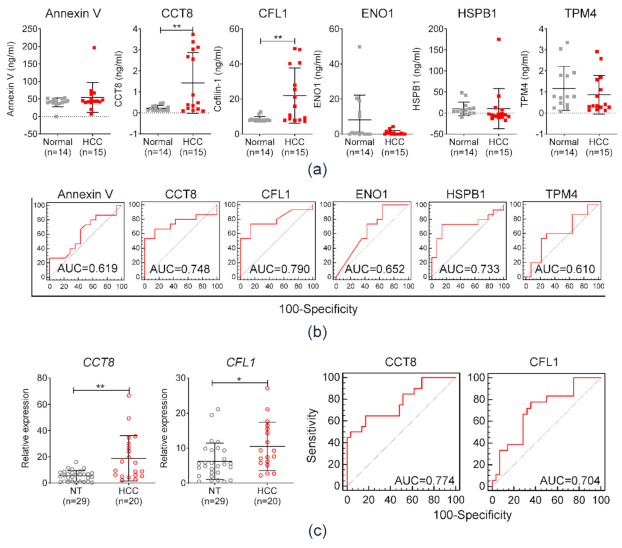
Diagnostic efficiency of annexin V, CCT8, CFL1, ENO1, HSPB1, and TPM4 in diagnosing HCC in the test cohort comprised of normal healthy individuals (normal) and patients with HCC. (**a**) Comparison of the six protein expressions between 14 healthy individuals (normal) and 15 patients with HCC. (**b**) Area under the curve (AUC) and receiver operating characteristics (ROC) of six protein markers in diagnosing HCC. (**c**) Left panel: relative expression of CCT8 and CFL1 in serum exosomal mRNA of 29 healthy individuals (normal) and 20 patients with HCC. Right panel: area under the curve (AUC) and receiver operating characteristics (ROC) of serum exosomal CCT8 and CFL1 expression.

Diagnostic efficiencies of the selected exosomal proteins were evaluated in a test cohort comprised of 14 healthy individuals and 15 patients with HCC. The serum concentrations of six candidates were measured by ELISA. The levels of expression of CCT8 and CFL1 were significantly higher in patients with HCC as compared to those of the control group (** *p* < 0.01; Figure 4a). The AUCs of each serum protein used to diagnose HCC were 0.619, 0.748, 0.790, 0.652, 0.733, and 0.610, corresponding to ANAX5, CCT8, CFL1, ENO1, HSPB1, and TPM4, respectively (Figure 4b). Among them, serum CCT8 and CFL1 exhibited the highest AUCs and showed significantly higher levels in patients with HCC. In addition to protein expression, mRNA expression of serum exosomal CCT8 and CFL1 was evaluated by qRT-PCR. Figure 4c (right panel) displays serum exosomal mRNA expression levels of CCT8 and CFL1. Concordant with serum protein levels, mRNA expression of serum exosomal CCT8 and CFL1 was significantly higher in HCC patients. Figure 4c (right panel) demonstrates the AUC of HCC diagnosis according to serum exosomal CCT8 and CFL1 expression values. The corresponding AUCs of serum exosomal CCT8 and CFL1 were calculated as 0.774 and 0.704, respectively. Consequently, CCT8 and CFL1 were selected for further study in an independent validation cohort.

### 3.4. Clinical Relavance of Serum Protein Markers in the Validation Cohort

**Figure 5:**
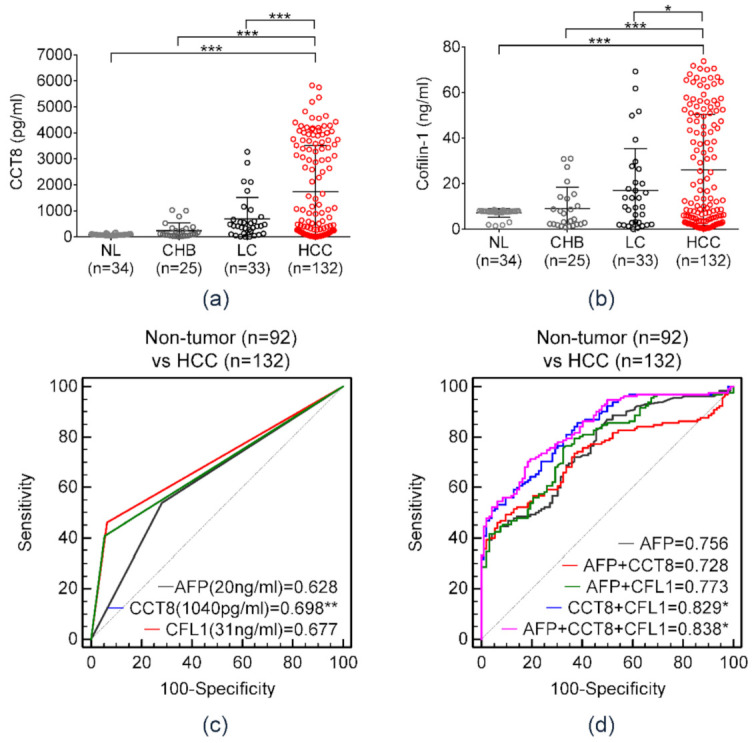
Diagnostic significance of serum AFP, CCT8, and CFL1 in diagnosing HCC in the validation cohort comprised of 34 normal individuals, 25 patients with chronic hepatitis B, 33 patients with liver cirrhosis, and 132 patients with HCC. (**a**,**b**) Expression level of serum (**a**) CCT8 and (**b**) CFL-1 according to clinical liver disease status. (Welch’s *t*-test; * *p* < 0.05, ** *p* < 0.01, *** *p* < 0.001) (**c**) AUC of serum AFP and two serum protein markers in diagnosing HCC. (Welch’s *t*-test; ** *p* < 0.01) (**d**) Diagnostic performance of serum protein panels for hepatocellular carcinoma in the validation cohort. (Welch’s *t*-test; * *p* < 0.05).

Table 1 shows baseline characteristics of the validation cohort. The cohort consisted of 224 patients, which included 34 normal healthy individuals (control), 25 patients with CHB, 33 patients with LC, and 132 patients with HCC. Figure 5a,b shows the serum CCT8 and CFL1 expressions according to liver disease status. Serum CCT8 and CFL1 gradually increased according to progression of clinical liver disease status. Figure 5c displays the AUCs of each serum marker for HCC diagnosis in the validation cohort. Serum CCT8 demonstrated the highest AUC among the three serum markers, and it was significantly higher than that of serum AFP. The AUC of serum CCT8 was calculated as 0.698 at a cut-off value of 1.04 ng/mL, whereas the AUC of serum AFP was measured as 0.628 at a cut-off value of 20 ng/mL. The AUC of serum CFL1 was measured as 0.677 at a cut-off value of 31 ng/mL (Table 2).

Further analysis was performed to assess AUCs according to the combination of serum markers (Figure 5d, Table 3). The combination of the three markers demonstrated the highest AUC (AUC = 0.838, 95% confidence interval (CI) = 0.773–0.876), with 70.46% sensitivity and 81.52% specificity. The AUC of the combination of serum CCT8 and CFL1 was calculated as 0.829 (95% CI = 0.773–0.876, sensitivity = 85.61%, specificity = 61.96%). The combination of novel serum biomarkers showed a significantly higher AUC than that of serum AFP, which is used as a conventional serum marker in HCC diagnosis (* *p* < 0.05).

### 3.5. Prognostic Significance of Serum CFL1 and CCT8 in Patients With HCC

**Figure 6:**
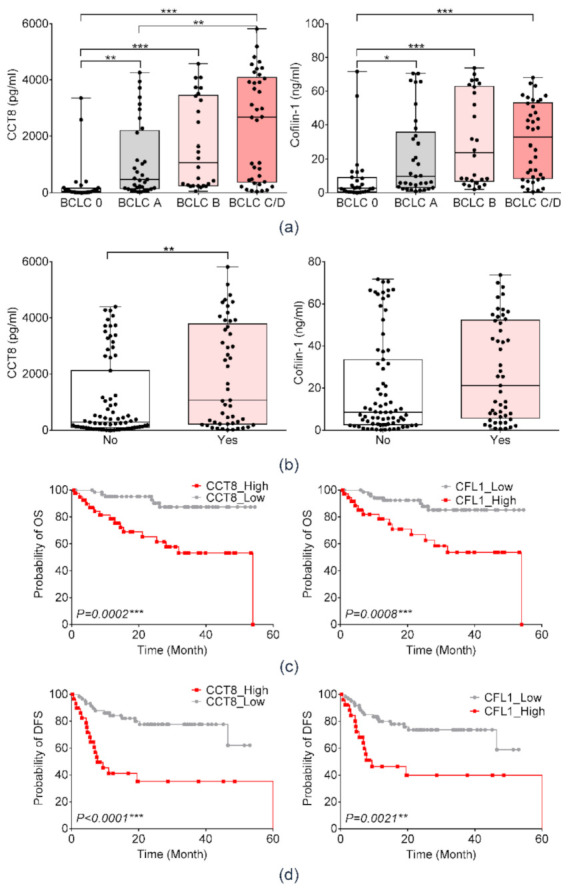
Prognostic significance of serum CCT8 and CFL1 in patients with HCC among the Ajou University Hospital liver disease cohort. (**a**) Expression of serum CCT8 and CFL1 according to BCLC stage guidelines. (Welch’s *t*-test; * *p* < 0.05, ** *p* < 0.01, *** *p* < 0.001) (**b**) Expression of serum CCT8 and CFL1 according to presence of vascular invasion. (Welch’s *t*-test; ** *p* < 0.01) (**c**) Overall survival according to expression of serum CCT8 and CFL1 in the validation cohort. (**d**) Disease-free survival according to expression of serum CCT8 and CFL1 in the validation cohort.

We also evaluated the prognostic implications of serum CFL1 and CCT8 in patients with HCC. The serum concentration of CCT8 and CFL1 gradually increased significantly with advancement in BCLC stage in patients with HCC (Figure 6a). Serum CCT8 was significantly higher in patients with vascular invasion as compared to patients without vascular invasion (*p* < 0.001). The serum concentration of CFL1 was higher in patients with vascular invasion; however, it was not statistically significant (Figure 6b).

The Kaplan–Meier survival analyses were performed to evaluate DFS and OS according to serum levels of CCT8 and CFL1 (Figure 6c,d). Patients with higher serum CCT8 showed poor DFS (*p* = 0.0002) and poor OS (log rank, *p* < 0.0001). Additionally, higher serum CFL1 was significantly associated with poor DFS (*p* = 0.0008) and OS (log rank, *p* = 0.0021).

## 4. Discussion

Conventionally, tissue acquisition is essential for acquiring genetic and proteomic information on solid tumors. Although most cancers are diagnosed by pathological confirmation, HCC can be diagnosed by typical radiological findings without pathological diagnosis [17,18]. Therefore, obtaining molecular tumor information from patients with HCC who underwent non-surgical treatment strategies is somewhat difficult.

Liquid biopsy is an emerging technology for detecting tumor-derived molecules by analyzing circulating tumor molecules in body fluid such as blood [16,19], and it could be a useful alternative collection method instead of solid tissue biopsy. Recent studies show that cancer cell-derived exosomes play an important role by delivering oncogenic molecules from tumor cells to other neighboring cells [20]. Therefore, exosomal contents have been studied as a major target of liquid biopsy for discovering diagnostic and therapeutic targets. Among the various exosomal contents, exosomal proteins are highlighted as potential biomarkers in different type of cancers [21,22,23]. Here, we identified proteins specifically overexpressed in HCC cell-derived exosomes compared to that of normal hepatocytes and investigated their use as clinical biomarkers. Fifteen overexpressed exosomal proteins derived from HCC cells were identified by performing proteomic analyses. Among them, six proteins, CCT8, CFL1, ENO1, TPM4, ANXA5, and HSPB1, were selected as biomarker candidates based on diagnostic and prognostic implications from several public-omics databases. Using the validation cohort, we demonstrated that serum CCT8 and CFL1 could serve as potential diagnostic and prognostic biomarkers for patients with HCC.

Previously, there has been only one study that investigated overexpressed proteins in HEX. The authors demonstrated many overexpressed oncogenic molecules in HEX [23] and revealed that HEX enhanced the migratory and invasive properties of normal hepatocytes via PI3K/AKT and MAPK signaling pathways. Although the previous in vitro study validated oncogenic potential and molecular mechanisms of HEX in hepatocarcinogenesis, we focused on the clinical significance of HEX-overexpressed proteins as biomarkers in a real-world clinical cohort. Cancer cell-derived exosomal proteins have been highlighted as a promising non-invasive biomarker in various cancers. For example, Melo et al. reported that pancreatic cancer cell-derived exosomes were enriched with glypican-1, and that glypican-1-positive circulating exosomes could be a potential diagnostic biomarker for early-stage pancreatic cancer [24]. In breast cancer, proteomic analyses revealed that developmental endothelial locus-1 (DEL-1) was enriched in plasma from patients with breast cancer as compared to normal individuals, and the diagnostic accuracy of exosomal DEL-1 for breast cancer was excellent with an AUC of 0.961 [25]. However, there are no studies focusing on the clinical implication of exosomal proteins from HCC as circulating biomarkers. In the present study, CCT8 and CFL1, overexpressed HEX proteins, were identified as potential serum diagnostic biomarkers in patients with HCC. They were also strongly associated with advanced tumor stage, vascular invasion, poor DFS, and poor OS.

Our study has several limitations. First, pathogenetic mechanisms of CCT8 and CFL1 in hepatocarcinogenesis were not evaluated. However, many other previous studies have already reported the oncogenic potential of CCT8 and CFL1 in various types of cancers. For instance, CCT8 promotes HCC cell proliferation and metastasis via the GRP94/CCT8/c-Jun/EMT-signaling cascade [26,27]. CFL1 is reported as a terminal effector involved in cytoskeletal rearrangement. Furthermore, it has been suggested as a therapeutic target and prognostic biomarker in various types of cancers including HCC [28,29,30,31,32,33]. Xu et al. reported that HBx-induced CFL1 accumulation could play an important role in development of HCC [34]. Second, although a validation study was performed with an internal patient cohort at our institution, additional external validation studies should be performed with larger patient cohorts to validate the result of our study.

## 5. Conclusions

In conclusion, we demonstrated proteins specifically overexpressed in HEX and their clinical significance as potential serum biomarkers. Serum CCT8 and CFL1, overexpressed proteins in exosomes derived from HCC, were identified as promising diagnostic and prognostic biomarkers for patients with HCC.

## Figures and Tables

**Table 1:** Baseline characteristics of patients in validation cohort (*n* = 224).

Variables	Validation Cohort			
	Normal (*n* = 34)	CHB (*n* = 25)	LC (*n* = 33)	HCC (*n* = 132)
Age (years), mean ± SD	34.2 ± 7.5	45.2 ± 11.2	53.0 ± 9.9	55.2 ± 9.02
Male sex, n (%)	4 (11.8)	18 (72)	19 (57.6)	109 (74.1)
AST, IU/ml	19.60 ±5.46	50.44 ± 51.40	79.19 ± 100.91	71.57 ± 96.77
ALT, IU/ml	20.00 ± 15.12	57.26 ± 66.30	77.25 ± 100.99	48.12 ± 59.28
Platelet, ×109/L	290.40 ± 42.17	190.56 ± 44.38	123.35 ± 65.59	166.44 ± 84.17
AFP (ng/mL), mean ± SD	1.71 ± 0.76	17.55 ± 24.96	49.94 ± 104.09	4290.43 ± 14525.79
Etiology, n HBV/HCV/alcohol/others			28/3/2/0	108/7/4/13
Albumin (g/L), mean ± SD		4.56 ± 0.41	4.05 ± 0.53	4.28 ± 0.55
Bilirubin (mg/dL) mean ± SD		0.81 ± 0.32	1.05 ± 1.03	1.34 ± 3.53
INR, mean ± SD		1.23 ± 0.30	1.24 ± 0.11	1.25 ± 0.55
Modified UICC stage, I/II/III/IVa/Ivb, n (%)				26 (19.7)/32(23.8)/24(18.2)/45(34.1)/5(3.8)

CHB, chronic hepatitis B; LC, liver cirrhosis; HCC, hepatocellular carcinoma; AST, aspartate transaminase; ALT, alanine transaminase; AFP, alpha-fetoprotein; HBV, hepatitis B virus; HCV, hepatitis C virus; INR, international normalized ratio; UICC, Union for International Cancer Control.

**Table 2:** Diagnostic efficiency of serum exosomal protein markers and AFP in HCC diagnosis.

HCC vs. Non-Tumor (Normal, CHB, and LC)							
	*p* vs AFP	AUC	95% CI	Sensitivity (%)	Specificity (%)	PPV (%)	NPV (%)
AFP (>20 ng/mL)	1	0.628	0.651–0.691	53.79	71.74	73.12	51.97
CCT8 (>1040 pg/mL)	0.0095	0.698	0.634–0.758	46.21	93.48	58.93	91.05
CFL1 (>31 ng/mL)	0.0758	0.677	0.612–0.738	40.91	94.57	91.53	52.73

HCC, hepatocellular carcinoma; CHB, chronic hepatitis B; LC, liver cirrhosis; AFP, alpha-fetoprotein; CCT8, chaperonin-containing TCP1 subunit 8; CFL1, cofilin 1; AUC, area under the curve; CI, confidence interval; PPV, positive predictive value; NPV, negative predictive value.

**Table 3:** Combination of serum exosomal protein markers and AFP in HCC diagnosis.

HCC vs. Non-Tumor (Normal, CHB, and LC)								
	*p* vs. AFP	Cut-Off	AUC	95% CI	Sensitivity (%)	Specificity (%)	PPV (%)	NPV (%)
AFP + CCT8	0.169	0.6106	0.728	0.664–0.785	46.21	93.48	91.05	54.77
AFP + CFL1	0.444	0.4704	0.773	0.712–0.826	75.76	67.39	76.92	65.96
CCT8 + CFL1	0.045	0.4434	0.829	0.773–0.876	85.61	61.96	76.35	75.00
AFP + CCT8 + CFL1	0.027	0.5438	0.838	0.783–0.884	70.46	81.52	84.55	65.79

HCC, hepatocellular carcinoma; CHB, chronic hepatitis B; LC, liver cirrhosis; AFP, alpha-fetoprotein; CCT8, chaperonin-containing TCP1 subunit 8; CFL1, cofilin 1; AUC, area under the curve; CI, confidence interval; PPV, positive predictive value; NPV, negative predictive value.

## Data Availability

The datasets generated during and/or analyzed during the current study are available from the corresponding author on reasonable request.

## Supplementary Materials

The following are available online at https://www.mdpi.com/article/10.3390/diagnostics11071221/s1: Figure S1: Raw data of Western blot analysis to detect marker of extracellular vesicles in exosomes; Representative 2D proteomic maps of THLE-2, Hep3B, and Huh-7 cell lines; Table S1: Proteins identified by mass spectrometry; Table S2: Expression of the 15 proteins which were markedly overexpressed (≥2.5 times) in HCC cell-derived exosome rather than THLE2-derived exosomes in public gene expression data-sets.
