# Supplementary material for: Overexpressed Proteins in HCC Cell-Derived Exosomes, CCT8, and Cofilin-1 Are Potential Biomarkers for Patients with HCC
Source: Diagnostics (Basel). 2021 Jul 6;11(7):1221. doi: 10.3390/diagnostics11071221 (PMC8307801; doi:10.3390/diagnostics11071221)

Overexpressed Proteins in HCC cell-derived Exosomes, CCT8 and Cofilin-1, are Potential Biomarkers for Patients with HCC

Supplementary Table S1. Proteins identified by mass spectrometry

	Spot ID	Protein GI accession	DAVID gene symbol
Hep3B	151	gi28592	ALB
	228	gi582045552	ALB
	256	No significant hits to report	No significant hits to report
	344	No significant hits to report	No significant hits to report
	362	gi31676	GC
	589	gi902787	YWHAE
	597	gi136408	PRSS1
	598	No significant hits to report	No significant hits to report
	668	gi37637	ANXA5
	709	gi902787	YWHAE
	771	No significant hits to report	No significant hits to report
	856	gi1025735596	CFL1
	1100	No significant hits to report	No significant hits to report
	1126	gi287641	PRDX1
Huh7	325	gi35655	P4HB
	441	gi32478	HSPB1
	477	gi119339	ENO1
	480	gi119339	ENO1
	541	gi28252	ACTB
	543	gi28252	ACTB
	603	gi435476	KRT9
	668	gi37637	ANXA5
	685	gi37202	TPM4
	688	gi37403	Unnamed
	713	No significant hits to report	No significant hits to report
	732	gi28252	ACTB
	746	gi130348	PGAM1
	756	No significant hits to report	No significant hits to report
	805	No significant hits to report	No significant hits to report
	814	No significant hits to report	No significant hits to report
	839	gi435476	KRT9

	1100	No significant hits to report	No significant hits to report
	1105	gi30501	DEFA3
	1120	gi1136741	CCT8
	1122	No significant hits to report	No significant hits to report
	1125	gi186772	KRT1
THLE2	355	gi37492	TUBA1A
	356	gi37492	TUBA1A
	378	gi2674062	PHGDH
	379	gi37494	TUBB4B
	406	gi35959	TUBB4A
	433	gi263098	TBP
	545	No significant hits to report	No significant hits to report
	548	No significant hits to report	No significant hits to report
	557	gi30251	CLU
	571	No significant hits to report	No significant hits to report
	579	gi30251	CLU
	606	gi32015	TUBA4A
	630	No significant hits to report	No significant hits to report
	646	No significant hits to report	No significant hits to report
	717	gi37494	TUBB4B
	774	No significant hits to report	No significant hits to report
	776	gi190879	RAN
	1103	No significant hits to report	No significant hits to report

Supplementary Table S2. Expression of the 15 proteins which were markedly overexpressed (≥ 2.5 times) in HCC cell-derived exosome rather than THLE2-derived exosomes in public gene expression data-sets

`	TCGA_LIHC		ICGC_LIRI		GSE77314	
	Fold Changes	P value	Fold Changes	P value	Fold Changes	P value
CFL1	1.90	0.000	2.00	0.000	1.94	0.000
PRSS1	1.58	0.000	1.06	0.011	1.20	0.080
PRDX1	1.46	0.000	1.75	0.000	1.51	0.000
YWHAE	1.03	0.476	1.12	0.000	-1.03	0.305
GC	0.44	0.000	-2.10	0.000	-3.61	0.000
ANXA5	1.60	0.000	1.22	0.004	1.33	0.007
ENO1	1.65	0.000	1.61	0.000	2.49	0.000
TPM4	1.73	0.000	1.45	0.000	1.42	0.003
KRT1	0.70	0.074	1.00	0.834	-1.01	0.297
P4HB	1.52	0.000	1.68	0.000	1.43	0.000
PGAM1	1.04	0.583	1.22	0.000	1.15	0.125
CCT8	1.67	0.000	1.54	0.000	1.52	0.000
KRT9	1.12	0.021	1.01	0.000	1.00	0.083
DEFA3	0.34	0.000	-1.11	0.000	-1.13	0.047
HSPB1	3.50	0.000	2.83	0.000	3.08	0.000

Supplementary Figure S1. Representative 2D proteomic maps of THLE-2, Hep3B, and Huh-7 cell lines.

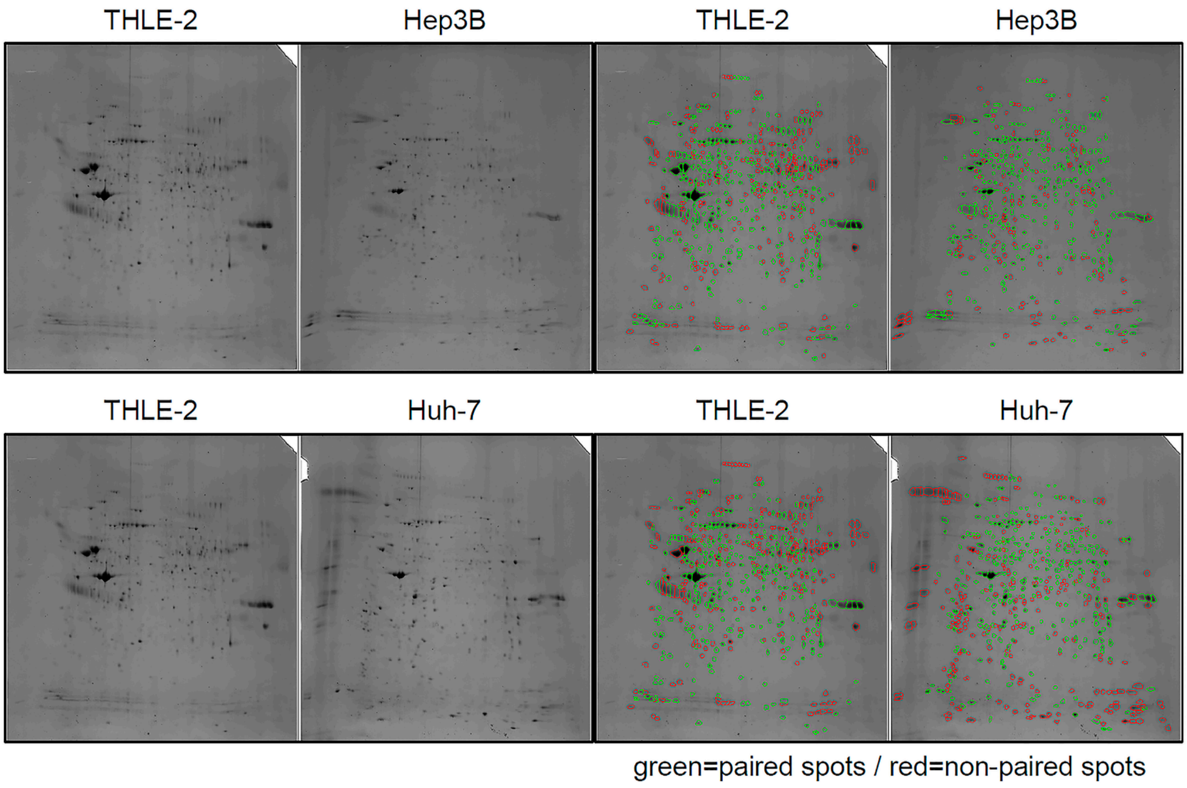

Supplement: Supplementary file 1 [file diagnostics-11-01221-s001.zip › diagnostics-1283622-supplementary.pdf]
